# Notes on Myotomy and Tenotomy

**Published:** 1841-10-16

**Authors:** Reynell Coates


### MEDICAL EXAMINER.

DEVOTED TO MEDICINE, SURGERY, AND THE COLLATERAL SCIENCES.

No. 42.] PHILADELPHIA, SATURDAY, OCTOBER 16, 1841. [Vol. IV

### ORIGINAL COMMUNICATION.

### NOTES ON MYOTOMY AND TENOTOMY.

BY REYNELL COATES, M. D.

To the Editors of the Medical Examiner.

Gentlemen:—By the review of the position of the foot at the conclusion of the second stage of the mechanical treatment, it will be perceived that the attitude has then become favourable to the application of mechanical forces designed chiefly to produce simple flexion by acting upon the contracted extensors. To make this a primary object at an earlier period—though such would seem to be the chief design of most the apparatus usually employed from the commencement—is obviously contrary to correct surgical principles. For, while the foot is in the position observed in what is termed the third stage of varus, (*see page* 647, ¶3,) and the effective traction tends necessarily in the anterior direction, we gain nothing by any shoe, or by hinges at the ankle, such as are seen in almost every instrument for varus, as well as the pes equinus. If, at this stage of the treatment, we attempt to accomplish mechanical flexion at the same time that we endeavour to drive or draw the anterior half of the foot forward, so as to disengage it from the inner side of the ossa calcis and astragalus, we must do so by causing our forces to act obliquely, or, in other words, at a mechanical disadvantage. This can only be effected at the expense of *an increase of force*—the very thing which the surgeon is most anxious to avoid, because, under all circumstances, the soft parts are called upon to bear quite as much pressure as is desirable, and are subjected to unavoidable irritation.

But, although it is improper to make extension a primary object in this stage of the treatment, we are always acting on the muscles of the calf in such a manner as to promote their elongation, whatever apparatus we may employ. As neither teachers nor students in the medical profession are usually so well informed on the principles of mechanics as it would be desirable that they should be, I will not detain your readers with the proof of a position about to be taken. It is founded mainly upon an obvious mathematical principle, and a regular demonstration would be unintelligible to those who are unfamiliar with such subjects, while it would be regarded as supererogatory by those who are better informed.

When the angular deformity at the middle of the tarsus is very great, it is impossible to act effectively in its amelioration by means of any machinery applied to the foot alone, without employing the instep, or, rather the upper part of the head of the astragalus, as a *point d'appuy*, and applying over it a degree of force which the integuments of that part are incapable of enduring.

For this, and other reasons, almost all the apparatus employed in the mechanical treatment of the third stage of varus has been extended to the leg by means of splints of some kind, connected with a shoe or other contrivance equivalent thereto. A portion, at least, of the flexing and abducting force is applied by employing the leg, and occasionally the thigh also, as *points d'appuy*; and it is impossible thus to draw the anterior part of the foot forwards without expending a considerable share of this portion of force upon the os calcis, to which it is transmitted by the new formed or contracted ligaments of the inner side of the tarsus, and through which it reaches the tendons of the extensors of the foot. But, in the misplaced position of the highly extended os calcis, (*see page* 616,) this bone cannot yield to flexion without undergoing some degree of abduction at the same time.

If, then, we devote our exclusive attention during the first stage of the treatment of varus, to the disentanglement of the ossa scaphoides and cuboides from the inner side of the ossa calcis and astragalus by the simplest means, we lose nothing, *except, perhaps a little unimportant time*, by our singleness of purpose; for, by the very necessity of the case, the means employed produce a gentle flexion and abduction of the whole foot.

During the second stage of the treatment, (*see page* 647, ¶5,) the requisite twisting force, which acts directly upon the plantar surface of the anterior portion of the foot and upon its outer edge, must also be so applied as to make the leg and thigh the principal *points d'appuy*, unless we resort to complicated apparatus *for the purpose of acting at a mechanical disadvantage*. Here, then, as in the former stage of treatment, we necessarily effect gentle flexion and abduction of the foot, without the necessity of making these motions a *primary* object in the construction of machinery.

It has always appeared to me that the neglect of these mechanical principles is among the chief causes of the difficulty and pain attendant upon the mechanical treatment, so vividly depicted by many of the tenotomists. The deformity attendant upon complete varus is such as to present at least three indications of cure, upon all of which surgeons have endeavoured to act simultaneously instead of consecutively. Hence, the extreme complexity of much of the machinery, and the undue suffering resulting from the action of multitudinous straps, bars, cushions, joints, screws, rachets, levers, springs, &c., which enter collectively into the structure of many contrivances for club-foot. If fulfilled *successively*, instead of concentaneously, neither of these indications demands any very great degree of complexity in the machine. And here let me make a remark, *en passant*, on the subject of *springs*. These are used freely by the mechanical inventors of apparatus for club-foot; but neither muscle nor ligament can long endure the unremitting action of a spring of any considerable extending force. The only reason why the pressure of the truss spring in hernia is borne so readily is, that the pressure is *lateral*—not longitudinal, and that, in most forms of the disease, it is made upon the surface of a large cavity, over which its action is in a great degree diffused by the yielding or semi-fluid nature of the contents. The very delicate spring employed in complete sets of artificial teeth, merely to raise the weight of the false gums and teeth of the upper jaw—the action of which should become a nullity when the mouth is widely opened—is yet sufficient to produce extreme fatigue of the masseter, the temporal, and the pterygoid muscles, until they become familiar with its presence; and a very little undue strength renders it perfectly intolerable, as every dentist will testify. Is it, then, reasonable to expect the extensors or abductors of the foot to endure the continuous action of such springs as are often seen attached to apparatus for club-foot? There is no necessity, in any instance, for the use of springs in club-foot; and it is unsurgical to attempt to compel any muscle to undergo an unceasing taxation upon its tonicity. Every function requires occasional rest, and the use of forces which call for perpetual exertion may very well prove intolerable, even if it should not end in paralysis of the organ.

If the views here expressed be correct, it is necessary, in all comparisons of results between the simple mechanical treatment, and the combination of tendon cutting with machinery, that the particular instrument employed in the former, as well as the latter, should be specified. Those reports in which the surgeon claims to have succeeded after tenotomy, in cases in which the "mechanical treatment" previously adopted had proved intolerable or useless—and who among the advocates of the knife have failed in making such reports?—are worth nothing whatever to those who would arrive at fair conclusions on this interesting subject; for, there are many splendid and highly lauded combinations of polished brass, steel and leather, designed for the treatment of club-foot, which are fitted only for a place in some great museum, as monuments of the ingenuity with which both physiological and mechanical laws *have been evaded by men of reputation!* I must protest against the condemnation of the purely mechanical treatment upon the strength of the failure of machinery, however beautiful, the joints of which are moved by rachets and keys placed in such locations as to have no relation whatever to the centre of motion of the joint to which they correspond—where, for instance, a simple pivot in the centre of a plantar splint is made to represent the motion of the line of joints at the middle of the tarsus!—where one set of cogs, working antero-posteriorly, is used as a substitute for the ankle-joint, though placed at the distance of an inch or two from the malleolus, and another acting at right angles with the former, is designed to follow the motions of the os calcis in forced adduction, though fixed some two or three inches above the ankle, on an adult leg! It is said;

" ——— bright names will hallow song!"

but there *is no name* sufficiently bright to hallow the application of such apparatus to "men of mortal mould." Experiments tried with such contrivances, in cases of confirmed varus, value not a straw in estimating the relative merits of tenotomy, and the treatment of club-foot without operation.

Let us return from this long digression.—Having no intention—here and at present—to enter the lists in argument with individuals, it would be useless to amplify on the subject. Another occasion may be sought for the critical analysis of special apparatus, whether open to the profession, *or concealed in favour of particular practitioners;* for there are many of both classes in the range of orthopœdic practice against which it might be desirable to enter a caviat, as the progress of science throws them under the inspection of the independent journalist.

Towards the conclusion of what I have termed the second stage of the mechanical treatment, when the foot is in the position described at the conclusion of the last note, its attitude is such as to render *flexion* the primary, and abduction, except so far as the os calcis alone is concerned, a secondary object. This results from the fact that it is impossible to apply any legitimate flexing force to the whole foot, when placed in a position approximating to that observed in simple pes equinus, without making the calf of the leg the principal *point d'appuy;* and it is equally impossible to apply flexing forces in this manner, without effecting gentle abduction upon a foot which still continues in a state of moderate adduction, or to avoid a diminution of the angular deformity at the centre of the tarsus, while the anterior extremity of the astragalus continues unnaturally prominent upon the instep. Our whole attention may therefore be given to flexion alone, from this point in the treatment, without fear of loss of time; and the future management of the case becomes even more simple than that required when the tendo Achilles has been previously divided; for, the angular deformity at the middle of the tarsus—the greatest obstacle to the removal of the deformity—has been already, in great degree, removed by previous mechanical measures, acting under superior mechanical advantages; *thus, actually saving instead of losing time.*

From this point the case is very much simplified, and our reasoning is narrowed down in such a manner that my remarks will be found, for the most part, equally applicable to "the first degree of varus," and the pes equinus. The necessary comments on the points of difference between these very similar deformities will be reserved for a future note.

In the condition of things to which reference has just been made, a foot-board or plantar splint comes into play, with some possible advantage, for the first time, and were I analysing the hundreds of forms of shoes and foot-pieces applied in the treatment of club-foot, this would be the place to discuss them. The angular deformity at the middle of the tarsus being very much diminished, it is now possible to employ the instep, or, rather, the parts over the anterior extremity of the astragalus, as a *point d'appuy;* and if these parts be drawn downwards by means of *well contrived* straps or other apparatus, to a foot-board, either simple or complex according to the nature of the deformity of the anterior part of the foot,—the said straps, &c. reacting upon a plantar splint—the little remaining angular deformity of the middle joint of the tarsus may be slowly overcome without much difficulty, and the unnatural exaggeration of the arch of the instep may be removed in a great degree, if not completely.

But it should be borne in mind that the upper part of the instep is incapable of enduring much long continued pressure; a capacity, in relation to which the various portions of the surface of the body differ very widely; and the difference has been by far too generally neglected by mechanical surgeons in consequence of their want of a mature knowledge of physiology. Most of the apparatus acting upon the instep in cases of club-foot is of a character to clasp or embrace the foot in a belt, upon the tightness of which the extension of the anterior half of the tarsus is made to depend. Even the most recent, and perhaps the most successful contrivances, lay open in some degree to this stricture, but the method is directly at war with both mechanical and physiological principles. Should Providence condemn all such inventors to the luxury of tight boots, and *the warmest gripe of friendship* from every acquaintance, until they become better acquainted with the resolution of forces, there would be no injustice in the decree!

The pressure of such bands becomes almost intolerable when it becomes decidedly efficient, and there is no necessity whatever for lateral compression of the tarsal bones in accomplishing the purpose in view. At best, pressure upon the instep should be regarded as a mere adjuvant, and not as a principal object, at this stage of the treatment, and we may consider the question of the future management of the case—for the present at least—as one of simple flexion of the foot, in which all the forces are brought to bear as directly as possible upon the contracted extensors. This subject will be discussed in my next.

